# Supplementary material for: Woronin bodies move dynamically and bidirectionally by hitchhiking on early endosomes in Aspergillus nidulans
Source: bioRxiv. 2023 Jan 21:2023.01.20.524968. Preprint. [Version 1] doi: 10.1101/2023.01.20.524968 (PMC9882315; doi:10.1101/2023.01.20.524968)
Supplement: Supplement 4 [file NIHPP2023.01.20.524968v1-supplement-4.pdf]

bioRxiv preprint doi: https://doi.org/10.1101/2023.01.20.524968; this version posted January 21, 2023. The copyright holder for this preprint (which was not certified by peer review) is the author/funder, who has granted bioRxiv a license to display the preprint in perpetuity. It is made available under aCC-BY 4.0 International license.

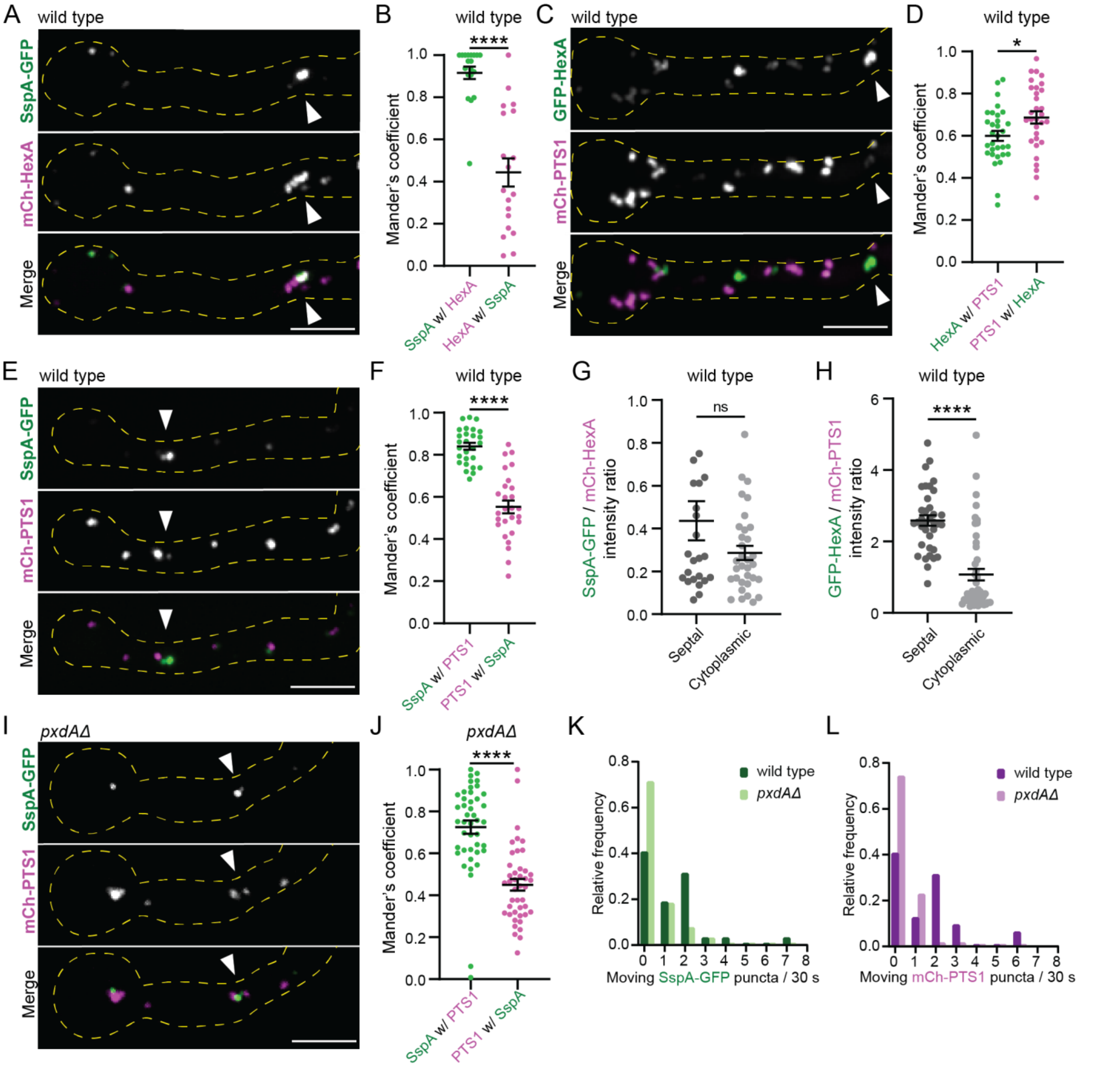

Figure S1. Woronin body and peroxisome colocalization and co-occurrence in whole germlings. (A, C, E, I) Example max-Z projections of colocalization between GFP and mCherry-tagged markers for Woronin bodies and peroxisomes in wild type (A, C, E) or *pxdAΔ* (I) germlings. White arrows indicate the septum closest to the spore head. Scale bars = 5 µm. **(B, D, F)** Average Mander's coefficient for each pair of markers in wild type germlings. B) SspA-mGFP5 and mCherry-HexA puncta in wild type (n = 19 germlings; mean ± SD: SspA w/ HexA = 0.92 ± 0.13; HexA w/ SspA = 0.44 ± 0.29). D) mGFP5-HexA and mCherry-PTS1 puncta in wild type (n = 31 germlings; mean ± SD: HexA w/ PTS1 = 0.60 ± 0.13; PTS1 w/ HexA = 0.69 ± 0.16) F) SspA-mGFP5 and mCherry-PTS1 puncta in wild type (n = 27 germlings; mean ± SD: SspA w/ PTS1 = 0.84 ± 0.085; PTS1 w/ SspA = 0.55 ± 0.16). **(G)** Average intensity ratio of SspA-mGFP5 and mCherry-HexA in puncta at septa and in the cytoplasm (n puncta, mean ± SD: septal = 24, 0.44 ± 0.45; cytoplasmic = 33, 0.29 ± 0.19). **(H)** Average intensity ratio of mGFP5-HexA and mCherry-PTS1 in puncta at septa and in the cytoplasm (n, mean ± SD: septal = 36, 2.59 ± 0.89; cytoplasmic = 47, 1.1 ± 1.1). **(J)** SspA-mGFP5 and mCherry-PTS1 puncta in *pxdAΔ* germlings (n = 43 germlings; mean ± SD: SspA w/ PTS1 = 0.73 ± 0.21; PTS1 w/ SspA = 0.45 ± 0.18). **(K-L)** Histograms of the relative frequency of all movements (retrograde and anterograde) for SspA-mGFP5 (K) and mCherry-PTS1 (L) in adult hyphae (n hyphal tips: wild type = 32; *pxdAΔ* = 64). **For B, D, F-H, and J:** unpaired t-test. Error bars represent SEM. P > 0.05 (labeled ns for "not significant," or not shown), P ≤ 0.05 (*), P ≤ 0.01 (**), P ≤ 0.001 (***), P ≤ 0.0001 (****).

bioRxiv preprint doi: https://doi.org/10.1101/2023.01.20.524968; this version posted January 21, 2023. The copyright holder for this preprint (which was not certified by peer review) is the author/funder, who has granted bioRxiv a license to display the preprint in perpetuity. It is made available under aCC-BY 4.0 International license.

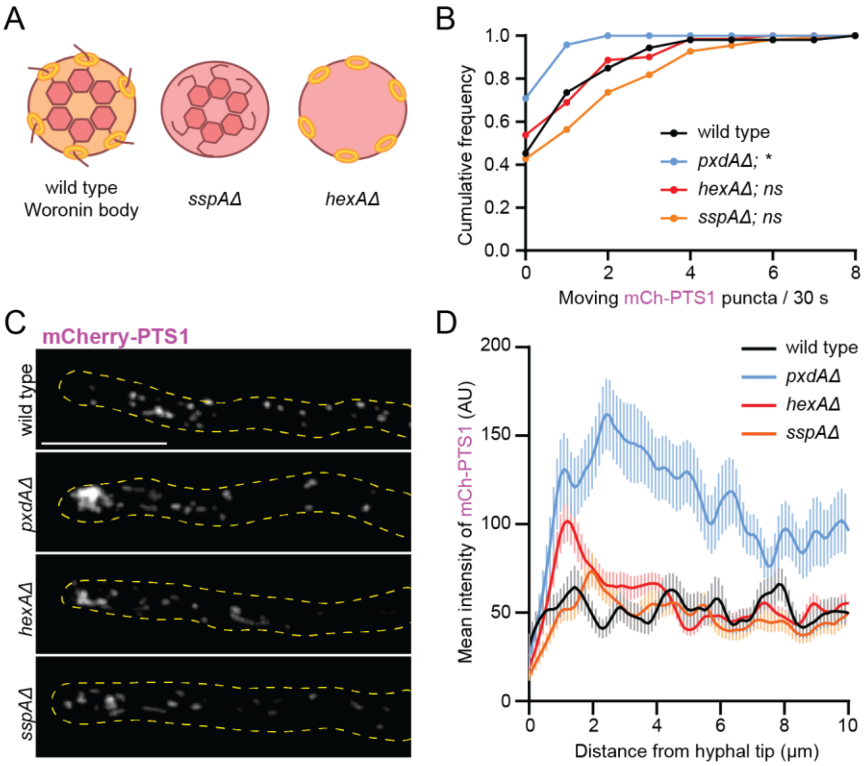

Figure S2. Peroxisome distribution and movement in cells lacking complete Woronin bodies. (A) Diagram of Woronin bodies lacking SspA or HexA. **(B)** Cumulative frequency plot of the number of moving mCherry-PTS1 puncta per 30 s in adult hyphal tips (n, mean ± SD puncta/30 s: wild type = 53 hyphae, 1.09 ± 1.5; *pxdAΔ* = 48 hyphae, 0.33 ± 0.56; *hexAΔ* = 132 hyphae, 1.02 ± 1.4; *sspAΔ* = 110 hyphae, 1.60 ± 1.86). The results of a Kruskal-Wallis with post-hoc Dunn's multiple comparisons test versus wild type are shown; $P > 0.05$ labeled ns for "not significant"; $P \leq 0.05$ (*). **(C)** Examples of mCherry-PTS1 in adult hyphal tips (max-Z projections). Scale bar = 10 µm. **(D)** Mean intensity of mCherry-PTS1 from the hyphal tip inwards (n: wild type = 33 hyphae; *pxdAΔ* = 30 hyphae; *hexAΔ* = 53 hyphae; *sspAΔ* = 54 hyphae). Error bars represent SEM.

bioRxiv preprint doi: https://doi.org/10.1101/2023.01.20.524968; this version posted January 21, 2023. The copyright holder for this preprint (which was not certified by peer review) is the author/funder, who has granted bioRxiv a license to display the preprint in perpetuity. It is made available under aCC-BY 4.0 International license.

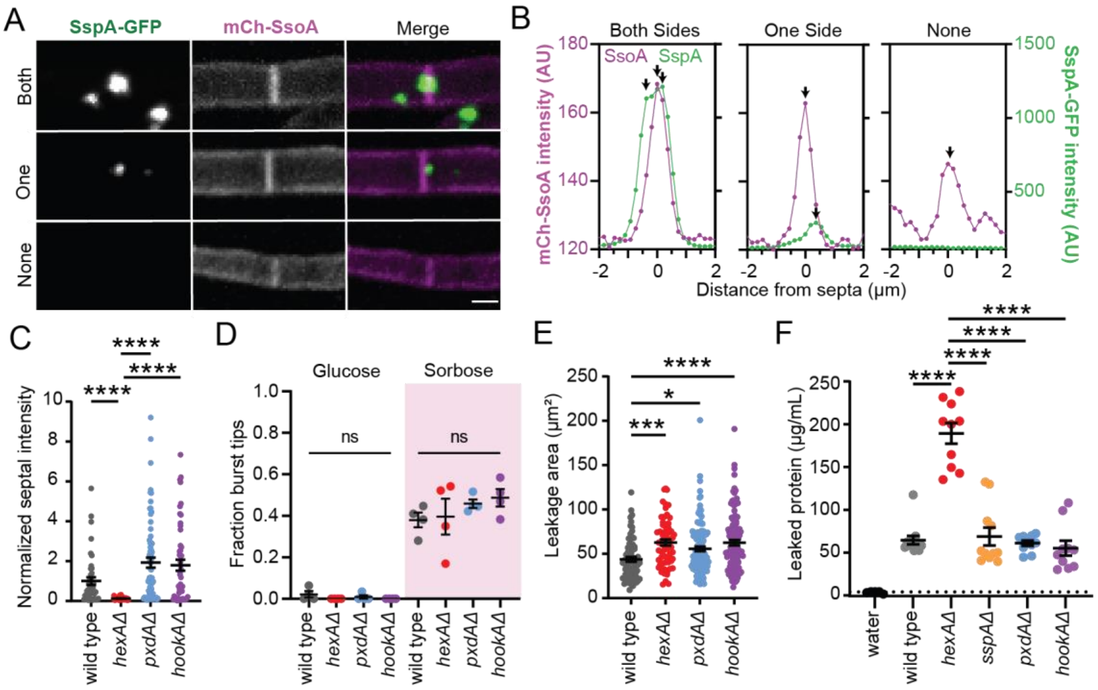

Figure S3. Woronin bodies at tip apical septa and cytoplasmic leakage assays. **(A)** Examples of Woronin body localization at septa. SspA-mGFP5 puncta were scored on max-Z projections as either present on both sides, one side, or none. Scale bar = 2 µm. **(B)** Line scans showing fluorescence intensity of Woronin bodies (SspA) or cell membrane at septa (SsoA) for each score category from A. Arrows indicate peak intensities for each fluorescent protein. **(C)** Normalized mean intensity of SspA-mGFP5 on either side (±0.5 µm) of apical septa for cells grown in glucose MM agar (n septa, mean ± SD AU: wild type = 43, 1.00 ± 1.24; *hexAΔ* = 45, 0.12 ± 0.6; *pxdAΔ* = 68, 1.93 ± 2.08; *hookAΔ* = 50, 1.80 ± 1.98). **(D)** Fraction of burst or damaged hyphal tips when grown in glucose or sorbose agar. Each circle represents the fraction burst hyphae from all tips imaged in a single experiment with 8-50 hyphae each (Glucose n experiments, mean ± SD: wild type = 4, 0.02 ± 0.03; *hexAΔ* = 4, 0 ± 0; *pxdAΔ* = 4, 0.01 ± 0.02; *hookAΔ* = 4, 0 ± 0. Sorbose n, mean ± SD: wild type = 4, 0.38 ± 0.07; *hexAΔ* = 4, 0.39 ± 0.17; *pxdAΔ* = 4, 0.46 ± 0.04; *hookAΔ* = 4, 0.49 ± 0.08. **(E)** Leakage area of cytoplasmic puddles for hyphae grown in sorbose (n burst hyphae, mean ± SD: wild type = 73, 43.7 ± 22.7 µm^2; *hexAΔ* = 63, 62.7 ± 26.8 µm^2; *pxdAΔ* = 125, 55.6 ± 28.9 µm^2; *hookAΔ* = 119, 62.5 ± 32.5 µm^2). **(F)** Quantification of cytoplasmic leakage after hypotonic shock. The amount of leaked protein in the supernatant was quantified via Bradford assay (n samples, mean ± SD: water = 5, -0.45 ± 1.3 µg/mL; wild type = 12, 62 ± 18 µg/mL; *hexAΔ* = 10, 189 ± 39 µg/mL; *sspAΔ* = 11, 66 ± 35 µg/mL; *pxdAΔ* = 10, 58 ± 28 µg/mL; *hookAΔ* = 10, 52 ± 28 µg/mL). **For C:** Kruskal-Wallis test with Dunn's multiple comparisons for nonparametric data. **For D-F:** Ordinary one-way ANOVA with post-hoc Tukey-Kramer test. **For C-F:** Error bars represent SEM. P > 0.05 (labeled ns for "not significant," or not shown), P ≤ 0.05 (*), P ≤ 0.01 (**), P ≤ 0.001 (***), P ≤ 0.0001 (****).

bioRxiv preprint doi: https://doi.org/10.1101/2023.01.20.524968; this version posted January 21, 2023. The copyright holder for this preprint (which was not certified by peer review) is the author/funder, who has granted bioRxiv a license to display the preprint in perpetuity. It is made available under aCC-BY 4.0 International license.

Movie S1 (separate file). Woronin body (SspA-mGFP5) and peroxisome (mCherry-PTS1) comigration in a wild type hyphal tip. A single Z plane image was taken every 300 ms via simultaneous dual-color spinning disk confocal microscopy (Nikon). The white arrow indicates a comigrating, bidirectional run. Scale bar = 10 µm.

Movie S2 (separate file). Woronin bodies (SspA-mGFP5) and peroxisomes (mCherry-PTS1) in a *pxdAΔ* hyphal tip. A single Z plane image was taken every 300 ms via simultaneous dual-color spinning disk confocal microscopy (Nikon). Scale bar = 10 µm.

Table S1 (separate file). PxdA (sheet 1) and DipA (sheet 2) BLASTp results for all 33 fungal species tested. The amino acid sequences used for each BLASTP search are shown in row 1 on both sheets.

bioRxiv preprint doi: https://doi.org/10.1101/2023.01.20.524968; this version posted January 21, 2023. The copyright holder for this preprint (which was not certified by peer review) is the author/funder, who has granted bioRxiv a license to display the preprint in perpetuity. It is made available under aCC-BY 4.0 International license.

Strain	Alias	Genotype	Source
RPA1256 RPA1257	SspA-GFP, mCherry-HexA	*[mCherry-HexA::Afpyro], pyroA4, [SspA-mGFP5::AfpyrG], pyrG89, nkuA::Bar*	This study
RPA1252 RPA1254	SspA-GFP, mCherry-PTS1	*yA::[gpdA(p)::mCherry-FLAG-PTS1::Afpyro], pyroA4, [SspA-mGFP5::AfpyrG], pyrG89, nkuA::bar*	This study
RPA1247 RPA1248	GFP-HexA, mCherry-PTS1	*yA::[gpdA(p)::mCherry-FLAG-PTS1::Afpyro], pyroA4, [mGFP5-HexA::AfpyrG], pyrG89, nkuA::bar*	This study
RPA1249 RPA1250	SspA-GFP, mCherry-PTS1, *pxdAΔ*	*yA::[gpdA(p)::mCherry-FLAG-PTS1::Afpyro], pyroA4, [SspA-mGFP5::AfpyrG], pyrG89, [pxdAΔ::Afribo], riboB2, pabaA1, nkuA::argB+*	This study
RPA1273	SspA-GFP, BFP-PTS1, PxdA-mKate	*wA::[gpdA(p)::2xtagBFP-PTS1::AfriboB], riboB2, [PxdA-mKate::Afpyro], pyroA4, [SspA-mGFP5::AfpyrG], pyrG89, nkuA:: argB+*	This study
RPA1326	SspA-GFP, mCherry-SsoA	*yA1, [SspA-mGFP5::AfpyrG], pyrG89, [mCherry-SsoA::Afpyro], pyroA4, pabaA1, nkuA::argB+*	This study
RPA1327	SspA-GFP, mCherry-SsoA	*[SspA-mGFP5::AfpyrG], pyrG89, [mCherry-SsoA::Afpyro], pyroA4, nkuA::argB+*	This study
RPA1320	SspA-GFP, mCherry-SsoA, *pxdAΔ*	*yA1, [SspA-mGFP5::AfpyrG], pyrG89, [mCherry-SsoA::Afpyro], pyroA4, [pxdAΔ::AfriboB], riboB2, pabaA1, nkuA::argB+*	This study
RPA1532	SspA-GFP, mCherry-SsoA, *hookAΔ*	*yA1, [SspA-mGFP5::AfpyrG], pyrG89, [mCherry-SsoA::Afpyro], pyroA4, [hookAΔ::AfriboB], riboB2, pabaA1, nkuA::argB+*	This study
RPA1321 RPA1322	SspA-GFP, mCherry-SsoA, *hexAΔ*	*yA1, [SspA -mGFP5::AfpyrG], pyrG89, [mCherry-SsoA::Afpyro], pyroA4, [hexAΔ::AfriboB], riboB2, pabaA1, nkuA::argB+*	This study
RPA1328 RPA1329	GFP, mCherry-SsoA	*yA1, wA::[mTagGFP2::AfpyrG], pyrG89, [mCherry-SsoA::Afpyro], pyroA4, pabaA1, nkuA::argB+*	This study
RPA1315 RPA1316	GFP, mCherry-SsoA, *pxdAΔ*	*yA1, wA::[mTagGFP2::AfpyrG], pyrG89, [mCherry-SsoA::Afpyro], pyroA4, pabaA1, [pxdAΔ::AfriboB], riboB2, nkuA::argB+*	This study
RPA1312 RPA1323	GFP, mCherry-SsoA, *hookAΔ*	*yA1, wA::[mTagGFP2::AfpyrG], pyrG89, [mCherry-SsoA::Afpyro], pyroA4, pabaA1, [hookAΔ::AfriboB], riboB2, nkuA::argB+*	This study
RPA1313 RPA1314	GFP, mCherry-SsoA, *hexAΔ*	*yA1, wA::[mTagGFP2::AfpyrG], pyrG89, [mCherry-SsoA::Afpyro], pyroA4, pabaA1, [hexAΔ::AfriboB], riboB2, nkuA::argB+*	This study
RPA1317 RPA1318	GFP, mCherry-SsoA, *sspAΔ*	*yA1, wA::[mTagGFP2::AfpyrG], pyrG89, [mCherry-SsoA::Afpyro], pyroA4, pabaA1, [sspAΔ::AfriboB], riboB2, nkuA::argB+*	This study
RPA528	GFP-RabA, mCherry-PTS1	*yA::[gpdA(p)-mCherry-FLAG-PTS1::Afpyro], pyroA4, [mTagGFP2-RabA::AfpyrG], pyrG89, riboB2, pabaA1, nkuA::argB+*	Tan *et al.*, 2014[39]
RPA861	GFP-RabA, mCherry-PTS1, *pxdAΔ*	*yA::[gpdA(p)-mCherry-FLAG-PTS1::Afpyro], pyroA4, [mTagGFP2-RabA::AfpyrG], pyrG89, [pxdAΔ::AfriboB], riboB2, pabaA1, nkuA::argB+*	Salogiannis *et al.*, 2016[39]
RPA1330 RPA1344	GFP-RabA, mCherry-PTS1, *hexAΔ*	*yA::[gpdA(p)-mCherry-FLAG-PTS1::Afpyro], pyroA4, [mTagGFP2-RabA::AfpyrG], pyrG89, [hexAΔ::AfriboB], riboB2, pabaA1, nkuA::argB+*	This study
RPA1345 RPA1346	GFP-RabA, mCherry-PTS1, *sspAΔ*	*yA::[gpdA(p)-mCherry-FLAG-PTS1::Afpyro], pyroA4, [mTagGFP2-RabA::AfpyrG], pyrG89, [sspAΔ::AfriboB], riboB2, pabaA1, nkuA::argB+*	This study

Table S2. Strains used in this study.

bioRxiv preprint doi: https://doi.org/10.1101/2023.01.20.524968; this version posted January 21, 2023. The copyright holder for this preprint (which was not certified by peer review) is the author/funder, who has granted bioRxiv a license to display the preprint in perpetuity. It is made available under aCC-BY 4.0 International license.

Plasmid ID	Plasmid Name	Source
RPB2188	*hookAΔ::AfriboB*	This study
RPB2007	*hexAΔ::AfriboB*	This study
RPB2184	*sspAΔ::AfriboB*	This study
RPB2042	*wA::mTagGFP2::AfpyrG*	This study
RPB2049	*SspA-mGFP5::AfpyrG*	This study
RPB2048	*mGFP5-HexA::AfpyrG*	This study
RPB2190	*mCherry-SsoA::AfpyroA*	This study
RPB564	*mCherry-HexA::AfpyroA*	This study

Table S3. Plasmids generated for this study.

bioRxiv preprint doi: https://doi.org/10.1101/2023.01.20.524968; this version posted January 21, 2023. The copyright holder for this preprint (which was not certified by peer review) is the author/funder, who has granted bioRxiv a license to display the preprint in perpetuity. It is made available under aCC-BY 4.0 International license.

Primer	Sequence (5'-3')	Purpose	Plasmid
LS65	AGCTTGGCGTAATCATGGTCATAGCTG	*F amplify pBH without overhangs*	all
LS66	CACTGGCCGTCGTTTTACAACGT	*R amplify pBH without overhangs*	all
JC192	AAAACGACGGCCAGTCATGCTTGCTTCCTCTTGCG	*F amplify upstream hookA*	RPB2188
LS119	cgattacatgaaggtccacctgtcatttgtcagCTTGAGCGGGCGCAGGG	*R amplify upstream hookA*	RPB2188
LS120	CGCCCCTGCGCCCGCTCAAGctgacaaatgacaggtggaccttcatgtaa	*F amplify AfriboB*	RPB2188
LS121	GGGTCATTTGTCAAGATTGAGCATGatgagtgtgacgagcatacaccgtg	*R amplify AfriboB*	RPB2188
LS122	cggtgtatgctcgtcacactcatCATGCTCAATCTTGACAAATGACCCCT	*F amplify downstream hookA*	RPB2188
JC96	GAAACAGCTATGACCATGATTACGCCAAGCAATAACTGTTGAAGGA-GATCCTGACAGTGG	*R amplify downstream hookA*	RPB2188
JC202	CATGCTTGCTTCCTCTTGCGCTCGC	*F targeting primer*	RPB2188
JC118	AATAACTGTTGAAGGAGATCCTGAC	*R targeting primer*	RPB2188
JC938	GTCACGACGTTGTAAAACGACGGCCAGTGTAGCACTTCGCACGTCGAACG	*F amplify upstream hexA*	RPB2007
JC939	cgagccagactcctgaacggcctcttCTTGCGAGCCTTGGGGTGATCTAC	*R amplify upstream hexA*	RPB2007
JC940	GTAGTAGATCACCCCAAGGCTCGCAAGaagaggccgttcaggagtctgg	*F amplify AfriboB*	RPB2007
JC941	AGGTCGTTTTAGAGAACCCCTGTAGACAGCatgagtgtgacgagcatacaccgtg	*R amplify AfriboB*	RPB2007
JC942	gtcgacacggtgtatgctcgtcacactcatGCTGTCTACAGGGGTTCTCTAAAACGACC	*F amplify downstream hexA*	RPB2007
JC915	GAAACAGCTATGACCATGATTACGCCAAGCTCACCTGAACGATCCAAC-GGTTTCGACG	*R amplify downstream hexA*	RPB2007
JC950	TAGCACTTCGCACGTCGAACGG	*F targeting primer*	RPB2007
JC953	CACCTGAACGATCCAACGGTTTCGACG	*R targeting primer*	RPB2007
LS11	CGACGTTGTAAAACGACGGCCAGTGtggaaagacgccatatctgggtctg	*F amplify upstream sspA*	RPB2184
LS26	AGCCAGACTCCTGAACGGCCTCTTaggaaaagcaatgaacaggaaacgcg	*R amplify upstream sspA*	RPB2184
LS27	ttcgcgtttcctgttcattgcttttcctAAGAGGCCGTTCAGGAGTCTGG	*F amplify AfriboB*	RPB2184
LS83	ACGGTGTATGCTCGTCACACTCATgaccaaaatggtccgtgctatcccat	*R amplify AfriboB*	RPB2184
LS29	TATGCTCGTCACACTCATaggatgtaatttcctcccggacaaatgatgac	*F amplify downstream sspA*	RPB2184
LS20	AGGAAACAGCTATGACCATGATTACGCCAAGCTgcggaggcggctcgacg	*R amplify downstream sspA*	RPB2184
LS137	tggaaagacgccatatctgggtctgg	*F targeting primer*	RPB2184
LS38	gcggaggcggctcgac	*R targeting primer*	RPB2184
JC192	AAAACGACGGCCAGTCATGCTTGCTTCCTCTTGCG	*F amplify upstream hookA*	RPB2582
LS628	actgtattattatattcagttatataagcgccggCTTGAGCGGGCGCAGG	*R amplify upstream hookA*	RPB2582
LS630	ccggcgcttatataactgaatataat	*F amplify AfpabA*	RPB2582
LS631	ttggaatgtttgcagcagc	*R amplify AfpabA*	RPB2582
LS629	acttgctgctgcaaacattccaaCATGCTCAATCTTGACAAATGACCCCT	*F amplify downstream hookA*	RPB2582
JC96	GAAACAGCTATGACCATGATTACGCCAAGCAATAACTGTTGAAGGA-GATCCTGACAGTGG	*R amplify downstream hookA*	RPB2582
JC202	CATGCTTGCTTCCTCTTGCGCTCGC	*F targeting primer*	RPB2582
JC118	AATAACTGTTGAAGGAGATCCTGAC	*R targeting primer*	RPB2582
JC165	AGTCACGACGTTGTAAAACGACGGCCAGTGCTCTGGAACAGTCTCGCCG	*F amplify upstream wA*	RPB2042
JC4	TGATTCTATAGGAAGATCCAGGCACCGGTCGATCAGGAGAAGGAGAG-TCAAGTCCGCTGA	*R amplify upstream wA*	RPB2042
JC3	TCAGCGGACTTGACTCTCCTTCTCCTGATCGACCGGTGCCTGGATCTTCC-TATAGAATCA	*F amplify gpdA promoter*	RPB2042
JC745	CAATTCTTCACCTCCTGACATTGTGATGTCTGCTCAAGCGGGG	*R amplify gpdA promoter*	RPB2042
JC743	CGCTTGAGCAGACATCACAATGTCAGGAGGTGAAGAATTGTTCG-CAGGTATTGTTCCTGT	*F amplify mTagGFP2::AfpyrG::downstream wA*	RPB2042
wA DS R	GAAACAGCTATGACCATGATTACGCCAAGCTACTCG-GAAAGGGTGGTGAATGCGGCGTGC	*R amplify mTagGFP2::AfpyrG::downstream wA*	RPB2042
LS45	CTCTGGAACAGTCTCGCCGTCTTG	*F targeting primer*	RPB2042
LS46	ACTCGGAAAGGGTGGTGAATGCG	*R targeting primer*	RPB2042
LS21	TCACGACGTTGTAAAACGACGGCCAGTGtttccttgagctcaccgcgttc	*F amplify sspA CDS*	RPB2049
LS22	TGGCACCGGCTCCAGCGCCTGCACCAGCTCCccggtagtcgggtccaggg	*R amplify sspA CDS*	RPB2049
LS23	gagtacgagaagggccctggacccgactaccggGGAGCTGGTGCAGGCGC	*F amplify mGFP5*	RPB2049
LS24	ggaaattacatcctTTATTTGTATAGTTCATCCATGCCATGTGTAATCCC	*R amplify mGFP5*	RPB2049
LS25	GGATGAACTATACAAATAAaggatgtaatttcctcccggacaaatgatga	*F amplify sspA 3' UTR*	RPB2049
LS16	GCGAAGAGGGTGAAGAGCATTGtaaagaaataaagatgcaccgagcggcg	*R amplify sspA 3' UTR*	RPB2049
LS17	gccgctcggtgcatctttatttctttaCAATGCTCTTCACCCTCTTCGCG	*F amplify AfpyrG*	RPB2049
LS18	atgggatagcacggaccattttggtcCTGTCTGAGAGGAGGCACTGATGC	*R amplify AfpyrG*	RPB2049
LS19	GCATCAGTGCCTCCTCTCAGACAGgaccaaaatggtccgtgctatcccat	*F amplify downstream sspA*	RPB2049
LS20	AGGAAACAGCTATGACCATGATTACGCCAAGCTgcggaggcggctcgacg	*R amplify downstream sspA*	RPB2049
LS37	tttccttgagctcaccgcgttct	*F targeting primer*	RPB2049
LS38	gcggaggcggctcgac	*R targeting primer*	RPB2049
LS30	CGACGTTGTAAAACGACGGCCAGTGgaagatactcgaacctctgcccct	*F amplify upstream hexA*	RPB2048
LS31	ACTCCAGTGAAAAGTTCTTCTCCTTTCATcttgcgagccttggggtgatc	*R amplify upstream hexA*	RPB2048
LS32	ccccaaggctcgcaagATGAAAGGAGAAGAACTTTTCACTGGAGTTGTCC	*F amplify mGFP5*	RPB2048
LS33	taacccatTCCGGAGCCACTTCCTTTGTATAGTTCATCCATGCCATGTGT	*R amplify mGFP5*	RPB2048
LS34	ATGAACTATACAAAGGAAGTGGCTCCGGAatgggttactacgacgacgac	*F amplify hexA CDS + 3' UTR*	RPB2048
JC956	GGTATTTCAGACCCGCGAAGAGGGTGAAGAGCATT-Gacaggccctacatgcagccttatc	*R amplify hexA CDS + 3' UTR*	RPB2048
JC958	ttttactttttgataaggctgcatgtagggcctgtCAATGCTCTTCACCCTCTTCGCGG	*F amplify AfpyrG*	RPB2048
JC959	tttcgacccatacggtgccgagcagcgcgacctgcCTGTCTGAGAGGAGGCACTGATGCG	*R amplify AfpyrG*	RPB2048
JC960	GATAACAGCTTGGCATCACGCATCAGTGCCTCCTCTCAGA-CAGgcaggtcgcgctgctcg	*F amplify downstream hexA*	RPB2048
JC915	GAAACAGCTATGACCATGATTACGCCAAGCTcacctgaacgatccaacggtttcgacg	*R amplify downstream hexA*	RPB2048
LS39	ggaagatactcgaacctctgcccct	*F targeting primer*	RPB2048
LS40	cacctgaacgatccaacggtttcga	*R targeting primer*	RPB2048
LS77	AGTCACGACGTTGTAAAACGACGGCCAGTGcaccatgttgggggtc	*F amplify upstream ssoA*	RPB2190
LS107	tcctcgcccttgctcaccatCGTGAAGATATAATGGAATCAATGAATCAA	*R amplify upstream ssoA*	RPB2190
LS103	TGATTCATTGATTCCATTATATCTTCACGatggtgagcaagggcgaggag	*F amplify mCherry*	RPB2190
LS104	CACCGGCTCCAGCGCCTGCACCAGCTCCcttgtacagctcgtccatgccg	*R amplify mCherry*	RPB2190
LS108	gccactccaccggcggcatggacgagctgtacaagGGAGCTGGTGCAGGC	*F amplify ssoA CDS + 3' UTR*	RPB2190
LS124	AGCATCTGATGTCCgtaacaaaaagatattaagattattaggtgatgact	*R amplify ssoA CDS + 3' UTR*	RPB2190
LS125	tcttaatatctttttgttacGGACATCAGATGCTGGATTACTAAGGTAAT	*F amplify AfpyroA*	RPB2190

(continued next page)

bioRxiv preprint doi: https://doi.org/10.1101/2023.01.20.524968; this version posted January 21, 2023. The copyright holder for this preprint (which was not certified by peer review) is the author/funder, who has granted bioRxiv a license to display the preprint in perpetuity. It is made available under aCC-BY 4.0 International license.

Primer	Sequence (5'-3')	Purpose	Plasmid
LS126	gattagattatatatacccaGCGAGTGTCTACATAATGAAGGACAAATGC	*R amplify AfpyroA*	RPB2190
LS123	TATGTAGACACTCGCtgggtatatataatctaatccacgttaacagctcc	*F amplify downstream ssoA*	RPB2190
JC925	AAACAGCTATGACCATGATTACGCCAAGCTaagcgcggcgagagataagg	*R amplify downstream ssoA*	RPB2190
JC954	caccatgttgggggtcttggagtatc	*F targeting primer*	RPB2190
JC955	aagcgcggcgagagataagg	*R targeting primer*	RPB2190
K147	CGAACAGCGAGTTCGCGCG	*F amplify upstream hexA*	RPB564
K148	GAGGGAGAAGTCAACCTCGTGGACATCAGATGCTGGATTACTAAGGTAAT	*R amplify upstream hexA*	RPB564
S669	GGACATCAGATGCTGGATTAC	*F amplify AfpyroA*	RPB564
S670	CTTCATTATGTAGACACTCGC	*R amplify AfpyroA*	RPB564
K149	CATTTGTCCTTCATTATGTAGACACTCGCTAGCACTTCGCACGTCGAACG	*F amplify hexA promoter*	RPB564
K150	CACCCCAAGGCTCGCAAGATGGTGAGCAAGGGCGAGGAGGATAACA	*R amplify hexA promoter*	RPB564
K071	GTGAGCAAGGGCGAGGAGG	*F amplify mCherry*	RPB564
K152	GGCATGGACGAGCTGTACAAGGGAGCTGGTGCAGGCGCTG-GAGCCGGTGCC	*R amplify mCherry*	RPB564
K154	GGTGCAGGCGCTGGAGCCGGTGCCGGTTACTACGACGACGACGGTAACT	*F amplify hexA CDS + downstream*	RPB564
K155	GACACGAAACGAACGCCTCG	*R amplify hexA CDS + downstream*	RPB564
K157	CCGTCAACGAAGGGTATGGAAC	*F targeting primer*	RPB564
K156	CAGACAAGCGCGCGAGCTCC	*R targeting primer*	RPB564

Table S4. Primers used to make plasmids for this study.
